# Determination of citric acid pretreatment effect on nutrient content, bioactive components, and total antioxidant capacity of dried sweet potato flour

**DOI:** 10.1002/fsn3.747

**Published:** 2018-07-27

**Authors:** Chala G. Kuyu, Yetenayet B. Tola, Ali Mohammed, Hosahalli S. Ramaswamy

**Affiliations:** ^1^ Department of Postharvest Management Jimma University College of Agriculture and Veterinary Medicine Jimma Ethiopia; ^2^ Food Science and Agricultural Chemistry McGill University Ste. Anne de Bellevue QC Canada

**Keywords:** antioxidant, bioactive photochemical, drying, polyphenols, sweet potatoes

## Abstract

Orange flashed sweet potatoes are rich and inexpensive source of diet and antioxidants. The purpose of this study was to evaluate the effects of CA pretreatments and convective hot air drying temperature on proximate composition, bioactive components, and total antioxidant capacity of flour of five orange flashed sweet potato varieties. Moisture, protein, ether extract, ash, carbohydrate, fiber, β‐carotene, total phenolic compounds, and total antioxidant capacity in the dried flour samples were evaluated and reported in the range of 4.1–7.4%, 2.4–4.2%, 1.2–1.1.8%, 2.2–3.2%, 82.7–87.1%, 1.3–1.8%, 35.5–91.6 mg/100 g, 49.8–107.9 mg GAE/100 g, and 27.3–85.4%, respectively. The interaction effects of varieties, drying temperature, and CA concentration were significant (*p* ˂ 0.05) except for fiber. Kulto and SPK006/6/6 performed better for most of the parameters studied followed by SPK00/06. For almost all varieties, samples dried at 55°C and after treated in 3% CA solution had the highest percentage in terms of proximate composition, bioactive components, and total antioxidant capacities.

## 1 INTRODUCTION

Sweet potato [*Ipomoea batatas* (L.) Lam.] is a highly nutritious modified root crop rich in carbohydrate, only next to rice, corn, and cassava (Zuraida, 2003). It is a starch root, which contains high amounts of β‐carotene and amino acid (especially lysine) which is deficient in other cereal products like rice (Hassellund et al., 2013). Besides these, it also contains polyphones, which act as antioxidants to safeguard the human body from certain chronic diseases (Huang, Chang, & Shao, 2006). Recent research indicates that bioactive compounds such as polyphenols have many physiological benefits such as antioxidant, antiinflammation, blood vessel relaxation, and capillary wall‐stabilizing activities (Hassellund et al., 2013). They improve blood lipid profiles by increasing plasma high‐density lipoproteins (HDL) and decreasing the low‐density lipoproteins (LDL) (Qin et al., 2009). Being rich in carotenoids, total polyphenol content, and ascorbic acid, sweet potato is gaining importance as the least expensive source of antioxidants (Alam, Rana, & Islam, 2016).

White fleshed sweet potato (WFSP) variety is the staple food for 13 million people in the Southern Regional State of Ethiopia (Kurabachew, 2015). In contrast, the orange fleshed sweet potato (OFSP), known to be a good source of β‐carotene and energy (293 to 460 kJ/100 g), is easy to cultivate and fairly drought‐tolerant (Hagenimana et al., 2001). These characteristics make OFSP an excellent food and nutrition security crop to the region, but in terms of their nutrient content, bioactive components and antioxidant capacity are not characterized. Furthermore, despite its increasing importance as a valuable crop for food security, so far value addition attempts have not been conducted in terms of production of dehydrated product and minimization of the associated after‐harvest losses (Tiruneh, 2017).

The use of tuber crop in Ethiopia is limited, and it is consumed as an alternative carbohydrate source. This is generally performed with fresh tuber as postharvest storage or processing technology is not yet well developed. Dehydration could be an inexpensive technology that can be easily adapted to reduce the losses and improve its utilization in food formulations. Proper drying of OFSP can result in a stable product with better quality (Utomo, Man, Yaakob, Rahman, & Saad, 2008) when assisted with predrying treatments. Singh, Raina, Bawa, and Saxena (2003) used potassium metabisulfite and sodium chloride to improve the quality of chips from sweet potato. Ahmed, Akter, and Eun (2010) also used sodium hydrogen sulfite to improve the flour quality of OFSP. It has been shown that losses of scavenging ability, total phenolic contents, and degree of oxidation increase with increasing processing temperature and decrease when tuber is soaked in citric acid solution (Shih, Kuo, & Chiang, 2009). On the contrary, short heating reduces the activity of endogenous polyphenol oxidase which is responsible for oxidation of bioactive compounds (Ahmed et al., 2010). Therefore, this study aimed at to evaluate the effects of citric acid (CA) pretreatment and drying temperature on nutrient content, bioactive components, and antioxidant capacity of OFSP flours produced from different varieties.

## 2 MATERIALS AND METHODS

### 2.1 Sample collection and preparation

Five varieties (SPK00/06, SPK004/6/6, Guntute, Bucteca, and Kulto) of OFSP were collected from Jimma Agricultural Research Center. The roots were washed in tap water, and only those with uniform overall appearance, size, and shape were selected for the study. Tubers were then sliced into 1 mm size and dried for 8 hr (after preliminary work) after CA treatment (Ahmed, Akter, & Eun, 2011).

### 2.2 Experimental design and treatment combinations

Experiments were carried out using a completely randomized design having five sweet potato varieties (SPK00/06, SPK004/6/6, Guntute, Bucteca, and Kulto), with two CA treatments (1 and 3%) and two drying temperatures (55 and 65°C) in factorial arrangement, and replicated three times.

### 2.3 Data collected

#### 2.3.1 Proximate composition analysis

For evaluating the effect of treatment on the nutritional quality of the flour, all components of proximate composition were determined using standard analytical methods of AOAC (2005) (methods for moisture (925.09), dietary fiber (993.21), protein (960.52), fat (920.85), and ash (923.03)).

#### 2.3.2 Total polyphenol content

The total polyphenol contents were determined according to Blainski, Lopes, and De Mello (2013) which involved the reduction of Folin‐Ciocalteu reagent by phenolic compounds. Absorbances of prepared samples were measured at 765 nm using UV–Vis spectrophotometer (T80 Jiangsu, China). Gallic acid was used as the standard, and the total phenolic contents were expressed as mg of gallic acid equivalent (GAE) per g of sample (mg GAE/g sample).

#### 2.3.3 β‐Carotene determination

Extraction and determination of total β‐carotene were based on the method described in Park *(* 1987). After extraction, absorbance was read at 450 nm using UV–Vis spectrophotometer (T80 Jiangsu, China) and estimated against with concentration of β‐carotene standard curve (Sigma‐Aldrich).

#### 2.3.4 Determination of total antioxidant capacity and IC_50_ value

Antioxidant capacity was determined according to the method of Lu and Foo (2000) which involved DPPH (2,2‐diphenyl‐1‐picryl‐hydrazyl) free radical scavenging assay. Briefly, 10 g of sweet potato flour was mixed with 100 ml methanol and the mixture was homogenized for 1 min in a homogenizer (POLYTRON^®^ 2500E, Switzerland) and kept in a water bath at 20°C for 60 min. The samples were then centrifuged at 748 g for 15 min, and the supernatant was taken for analysis. The solvent extract of the sample was taken in 200, 400, 600, 800, and 1,000 μl concentrations in a test tube, and the volume was made up to 1 ml with the solvent and 2 ml of 0.1 mM DPPH was added to each tube. The mixture was shaken well and incubated at room temperature in the dark for 30 min. The decrease in absorbance of the resulting solution was then measured using UV–Vis spectrophotometer (T80 Jiangsu, China) at 517 nm. Scavenging activity was calculated from absorbance values of samples and control sample using the following equation:

$$\text{RSA}\;\left(\%\right)\;=\;\left(\frac{A_{c}-\frac{A_{t}}{A_{s}}}{A_{c}}\right)\;\times \;100$$

where RSA: radical scavenging activity; *A*_c_: absorbance of control; *A*_t_: absorbance of test solution; and *A*_s_: absorbance of standard solution.

The IC_50_ value, defined as the amount of the sample to scavenge 50% of the DPPH radicals, was calculated from percentage of radical scavenging activity results by plotting the graph of DPPH free radical scavenging activity versus concentration of the sample.

### 2.4 Statistical analysis

Statistical analysis was carried out using Minitab version 16 and analysis of variance (ANOVA) to determine the significance differences in nutritional, bioactive, and antioxidant contents of samples. Diagnostic tools like normal plot of residuals were tested prior to data analysis and indicated that the residuals of all parameters were normally distributed. Differences between the sample means were conducted using Tukey's test at α = 0.05 level for parameters showed significant difference.

## 3 RESULTS AND DISCUSSIONS

### 3.1 Proximate composition

Table 1 shows proximate compositions of different OFSP varieties treated in different concentrations of CA and dried at different drying temperatures. Moisture, ash, protein, ether extract, carbohydrate, and energy contents of the flour from dried slices were significantly (*p* < 0.05) affected by variety, CA concentration, and drying temperature.

**Table 1:** Mean values for proximate compositions (values in %) of OFSP pretreated with CA solutions and dried at different temperatures for five different varieties

CAC	Drying temperature (°C)	Varieties	MC	Ash	Protein	EE	Total carbo.	Energy (kcal/100 g)
CA (1%)	55°C	SPK00/06	7.4 ± 0.13^a^	3.15 ± 0.25^a^	3.4 ± 0.32^bcde^	1.8 ± 0.12^a^	82.8 ± 0.24^e^	360.9 ± 0.37 ^g^
		SPK004/6/6	7.2 ± 0.22^a^	2.4 ± 0.14 ^cd^	3.2 ± 0.16^cdef^	1.7 ± 0.14^ab^	84.2 ± 0.23^cde^	364.9 ± 0.43^efg^
		Guntute	6.0 ± 0.3^abcde^	2.8 ± 0.15^abcd^	3.1 ± 0.18^defh^	1.7 ± 0.13^ab^	84.2 ± 0.23^cde^	364.5 ± 0.36^efg^
		Bucteca	7.4 ± 0.21^a^	2.55 ± 0.15^bcd^	3.3 ± 0.12^cdef^	1.6 ± 0.14^abc^	83.9 ± 0.26^de^	363.3 ± 0.37 ^fg^
		Kulto	6.7 ± 0.33^ab^	2.9 ± 0.33^abc^	3.7 ± 0.14^bcd^	1.7 ± 0.15^ab^	83.7 ± 0.25^de^	365.0 ± 0.33^defg^
	65°C	SPK00/06	4.9 ± 0.23^cdef^	3.1 ± 0.12^ab^	2.4 ± 0.21 ^h^	1.2 ± 0.13^d^	86.6 ± 0.27^a^	367.2 ± 0.42^bcdef^
		SPK004/6/6	4.6 ± 0.13^ef^	2.2 ± 0.32^d^	3.0 ± 0.22^efh^	1.5 ± 0.13^bcd^	86.6 ± 0.25^a^	371.7 ± 0.33^ab^
		Guntute	5.4 ± 0.32^bcdef^	2.8 ± 0.14^abcd^	2.5 ± 0.32^gh^	1.4 ± 0.06 ^cd^	86.9 ± 0.24^a^	369.9 ± 0.37^abcd^
		Bucteca	4.7 ± 0.24^def^	2.5 ± 0.16 ^cd^	2.6 ± 0.14^fgh^	1.3 ± 0.07 ^cd^	87.1 ± 0.27^a^	370.6 ± 0.45^abc^
		Kulto	5.0 ± 0.32^cdef^	3.1 ± 0.17^ab^	3.3 ± 0.19^cdef^	1.2 ± 0.08^d^	85.7 ± 0.22^abc^	367.2 ± 0.33^bcdef^
CA (3%)	55°C	SPK00/06	6.3 ± 0.13^abcd^	3.1 ± 0.32^ab^	4.0 ± 0.12^ab^	1.8 ± 0.11^a^	83.3 ± 0.23^de^	365.4 ± 0.43^defg^
		SPK004/6/6	6.1 ± 0.33^abcde^	2.4 ± 0.34 ^cd^	3.9 ± 0.11^abc^	1.7 ± 0.14^ab^	84.5 ± 0.23^bcd^	368.9 ± 0.38^abcde^
		Guntute	6.4 ± 0.14^abc^	2.9 ± 0.13^abc^	4.1 ± 0.33^ab^	1.8 ± 0.15^a^	83.4 ± 0.22^de^	366.4 ± 0.37^cdef^
		Bucteca	6.7 ± 0.27^ab^	2.5 ± 0.16 ^cd^	3.8 ± 0.16^abcd^	1.8 ± 0.16^a^	83.8 ± 0.29^de^	366.8 ± 0.41^bcdef^
		Kulto	6.6 ± 0.35^ab^	3.2 ± 0.12^a^	4.2 ± 0.09^a^	1.8 ± 0.15^a^	82.7 ± 0.24^e^	363.8 ± 0.33 ^fg^
	65°C	SPK00/06	4.2 ± 0.16^f^	3.15 ± 0.14^a^	3.7 ± 0.33^bcde^	1.2 ± 0.13^d^	86.2 ± 0.23^a^	370.8 ± 0.34^abc^
		SPK004/6/6	4.2 ± 0.13^f^	2.4 ± 0.16 ^cd^	3.7 ± 0.33^bcd^	1.4 ± 0.11 ^cd^	86.5 ± 0.21^a^	373.6 ± 0.36^a^
		Guntute	4.1 ± 0.17^f^	2.9 ± 0.17^abc^	3.6 ± 0.33^bcde^	1.4 ± 0.11 ^cd^	86.3 ± 0.30^a^	371.8 ± 0.37^ab^
		Bucteca	4.1 ± 0.28^f^	2.4 ± 0.18 ^cd^	3.2 ± 0.33^cdef^	1.3 ± 0.12 ^cd^	87.1 ± 0.28^a^	372.9 ± 0.40^a^
		Kulto	4.2 ± 0.13^f^	3.0 ± 0.14^abc^	3.7 ± 0.33^bcd^	1.2 ± 0.07^d^	86.0 ± 0.27^ab^	370.4 ± 0.44^abc^
CV (%)			2.217	1.253	1.645	1.540	1.860	1.020

Notes

CAC = CA concentration; EE = ether extract; Carbo = carbohydrate. Results are mean values of triplicate determination, and means with different letters across the column are significantly different (*p* < 0.05).

#### 3.1.1 Moisture content

SPK00/06, SPK004/6/6, Bucteca, and Kulto varieties treated with 1% CA solution and dried at 55°C, and Bucteca and Kulto varieties treated with 3% CA solution and dried at 55°C had higher moisture contents (6.6–7.4%). The lowest values (4.1–4.6%) were associated with SPK00/06, SPK004/6/6, Bucteca, and Kulto treated with 3% CA and dried at 65°C, and SPK004/6/6 treated with 1% CA and dried at 65°C. There were also significant differences among the varieties with respect to moisture contents, and hence, the differences might also be associated with tissue morphological, structural, and chemical composition difference that would influence the migration of moisture. Results in this study showed maximum moisture content of 7.4%, which could result in a stable product for long‐term storage with reduced impact on product quality and stability.

#### 3.1.2 Ash content

After processing, ash content (2.8–3.15%) was highest for SPK00/06, Guntute, and Kulto, whereas the lowest (2.2–2.8%) was associated with SPK004/6/6 and Bucteca varieties. These variations probably associated with varietal differences as indicated in other studies (Van Hal, 2000; Woolfe 2008). It might be also due to the leaching effect of minerals in the CA solution during soaking. Different researchers have also reported that pretreatments such as soaking and blanching influenced the ash contents of sweet potato flour (Van Hal, 2000).

#### 3.1.3 Protein content

The highest mean value of protein (3.9–4.2%) was recorded from Kulto, SPK004/6/6, and Guntute varieties treated with 3% CA and dried at 55°C. The lowest (2.4–2.6%) was from SPK00/06, SPK004/6/6, Guntute, and Bucteca varieties treated in 1% CA and dried at 65°C. The variation observed in terms of protein contents may be due to varietal difference or nonenzymatic browning due to condensation of reducing sugars with amino groups due to Maillard reaction (Utomo et al., 2008). In terms of crude protein, the observed values are comparable with other root crops, such as cassava and yam (Grabowski, Truong, & Daubert, 2008). Although sweet potato is regarded as a high‐energy and low‐protein food, its protein in both fresh and flour forms has been reported to be of high biological value and could serve as a fairly important protein source among low‐income consumers (Van Hal, 2000).

#### 3.1.4 Ether extract

The highest ether extract (1.6–1.8%) was recorded from all varieties dried at 55°C and treated with both 1% and 3% CA concentrations, whereas the lowest (1.2–1.5%) was from all varieties dried at 65°C and treated with both 1% and 3% concentrations. The reduced fat content of the flour could be due to exposure to the drying conditions or fat oxidation with an increase in drying temperature. The fat contents observed in the present study were lower than reported in Padonou, Mestres, and Nago (2005) for cassava roots**.** The lower fat content associated with the sweet potato flour which would be desirable as the risk of oxidation is reduced thus preventing the development of off flavors during storage.

#### 3.1.5 Total carbohydrates

SPK00/06, SPK004/6/6, Bucteca, and Guntute varieties treated with 1% CA and dried at 65°C, and SPK00/06, SPK004/6/6, Bucteca, and Guntute varieties treated with 3% CA and dried at 65°C had the highest carbohydrate contents (86.2–87.1%). The results of carbohydrate contents of the flour were in close agreement with findings reported by different works (Adegunwa, Alamu, & Omitogun, 2011; Noman, Hoque, Haque, Pervin, & Karim, 2007). The total carbohydrate content in samples dried at 65°C was higher than in those dried at 55°C. This calculated value results from the lower values of all other proximate parameters as the content is calculated by difference. On the other hand, SPK00/06, SPK004/6/6, Bucteca, Guntute, and Kulto varieties dried at 55°C and treated with 1% CA, and Bucteca, Guntute, and Kulto varieties dried at 55°C and treated with 3% had the lowest carbohydrate contents (82.7–84.2%). These observed differences may also be associated with high moisture contents.

#### 3.1.6 Dietary fiber

**Figure 1:**
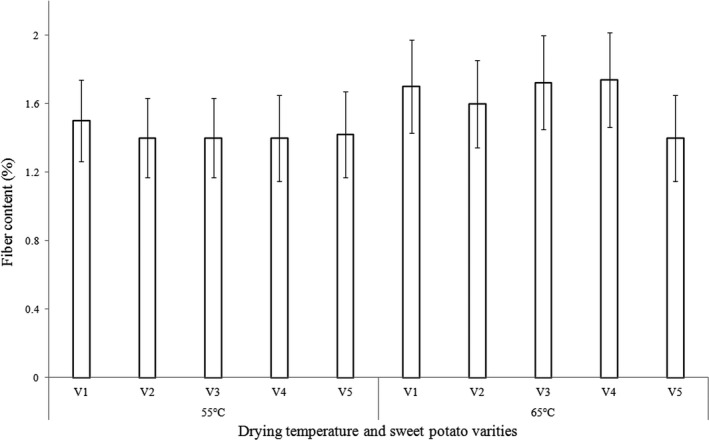
Two‐way interaction effect of fiber among variety and drying temperature (V1, V2, V3, V4, and V5 are SPK00/06, SPK004/6/6, Guntute, Bucteca, and Kulto varieties, respectively)

**Figure 2:**
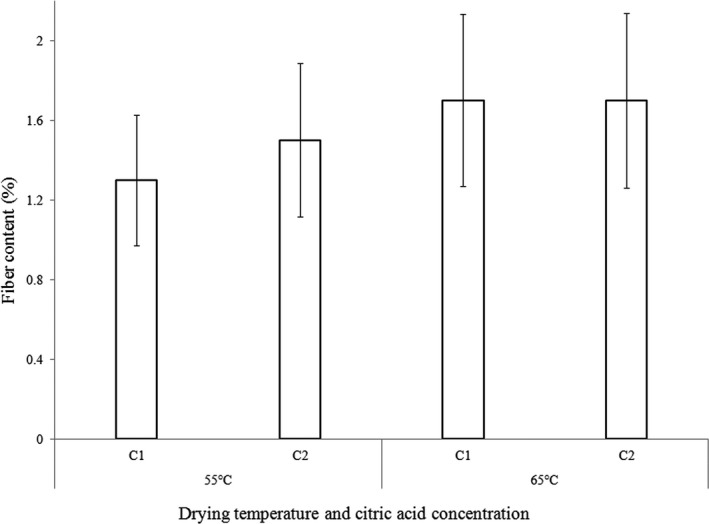
Two‐way interaction effect of fiber among CA concentration and drying temperature (V1, V2, V3, V4, and V5 are SPK00/06, SPK004/6/6, Guntute, Bucteca, and Kulto varieties, respectively)

The two‐way interaction effect of variety and drying temperature on fiber is shown in Figure 1, whereas Figure 2 shows the two‐way interaction effects of CA and drying temperature. The results of crude fiber obtained from this study (1.3–1.8%) were within the range of 1.01–3.87 g/100 g reported for 18 varieties of sweet potatoes in Hawaii, which is, however, less than what is reported in Sri Lanka (2.1 and 13.6%) on a dry matter basis (Senanayake, Ranaweera, Gunaratne, & Bamunuarachchi, 2013). These differences could be attributed to the processing method used in the present study which involved peeling of the relatively high fiber skins. Other researchers have reported low values compared to this study. Toledo and Burlingame (2006) reported 1.2 g/100 g and Rose and Vasanthakaalam (2011) reported 0.11–0.14% of the varieties studied in Rwanda.

Bucteca, Guntute, and Kulto varieties dried at 65°C had the highest fiber content (1.7–1.76%), whereas SPK004/6/6, Guntute, and Bucteca varieties dried at 55°C had the lowest fiber (1.4%). The results indicated that samples dried at 65°C with prior CA treatment (1% or 3%) had the highest fiber content and were significantly different from samples dried at 55°C. This may be due to the fact that high temperature could degrade other components and result in higher fiber. The characteristics between different varieties of the roots might also contribute to the observed differences.

#### 3.1.7 Gross energy

The results of the present study indicated that the interaction effect of varieties, CA concentration, and drying temperature showed the significant differences (*p* < 0.05). The calorific value was highest (373.6 kcal/100 g) in SPK004/6/6 variety treated with 3% CA and dried at 65°C. However, it is not statistically different from SPK00/06, Guntute, and Bucteca varieties treated with 3% CA and dried at 65°C, and SPK00/06, Guntute, Bucteca, and Kulto varieties treated with 1% CA and dried at 65°C.

### 3.2 Effect on bioactive compounds

#### 3.2.1 Total polyphenol content

The highest polyphenol contents (107.9 and 104 mg GAE/100 g) were recorded from SPK004/6/6 (107.9 mg GAE/100 g) and Kulto (104 mg GAE/100 g) varieties treated with 3% CA and dried at 55°C. The estimated lowest values were from SPK00/06 (49.8 mg GAE/100 g) and Bucteca (54.3 mg GAE/100 g) but treated with 1% CA and dried at 65°C, and Kulto and Bucteca varieties treated with 3% CA and dried at 65°C (Table 2). The polyphenol content of the flour was within the range of 51–171 mg GAE/100 g as reported for sweet potato flour (Yoshimoto, Okuno, Yamaguchi, & Yamakawa, 2001). The results in this study indicated that the highest concentration of CA contributed to the reduction in the oxidation rate of polyphenol compounds when combined with lower drying temperature. Polyphenol losses during the drying process were increased at 65°C (Perla, Holm, & Jayanty, 2012), and losses ranged from 24.4 to 53% during drying of sweet potato at 70°C. However, there are some contradictory results associated with the effect of temperature on the levels of polyphenols. Lavelli, Hippeli, Peri, and Elstner *(* 1999) stated that higher temperatures release more of the bound polyphenols due to breakdown of cell wall phenolic compounds. However, data reported in Ahmed et al. (2010) support the present finding which suggested that thermal processing had an adverse effect on the stability of polyphenols due to chemical degradation. Furthermore, varieties showed differences in polyphenol content, and among all studied varieties, SPK004/6/6 and Kulto showed better concentration of polyphenols as compared to others. Similar observations were made by Lachman et al. (2013), who stated that anthocyanins and total polyphenol contents vary with variety. Different researchers also stated that genetic factors may play an important role in the formation of secondary metabolites, including phenolic acids (Howard, Clark, & Brownmiller, 2003; Islam et al., 2002).

**Table 2:** Mean values for bioactive composition of OFSP flour pretreated at different CA concentrations and dried at different temperatures for five different varieties

CAC	Drying temperature (°C)	Varieties	Total polyphenol content (mg GAE/100 g)	β‐Carotene carotene (mg/100 g)
CA (1%)	55°C	SPK00/06	61.5 ± 0.11 ^g^	45.2 ± 0.07 ^g^
		SPK004/6/6	80.0 ± 0.12^d^	63.6 ± 0.11^d^
		Guntute	71.0 ± 0.14^e^	54.7 ± 0.16^e^
		Bucteca	64.6 ± 0.13 f^g^	48.2 ± 0.12 ^fg^
		Kulto	81.9 ± 0.14^d^	65.5 ± 0.17^d^
	65°C	SPK00/06	49.8 ± 0.15^i^	33.5 ± 0.11^i^
		SPK004/6/6	61.5 ± 0.14 ^g^	45.2 ± 0.12 ^g^
		Guntute	54.9 ± 0.12 ^h^	36.2 ± 0.08 ^hi^
		Bucteca	52.5 ± 0.12^hi^	38.5 ± 0.08^h^
		Kulto	61.7 ± 0.13 ^g^	45.3 ± 0.09 ^g^
CA (3%)	55°C	SPK00/06	98.9 ± 0.12^b^	82.6 ± 0.11^b^
		SPK004/6/6	107.9 ± 0.12^a^	91.6 ± 0.17^a^
		Guntute	90.3 ± 0.14^c^	74.0 ± 0.05^c^
		Bucteca	97.7 ± 0.13^b^	81.3 ± 0.08^b^
		Kulto	104.0 ± 0.12^a^	87.7 ± 0.12^a^
	65°C	SPK00/06	54.8 ± 0.11 ^h^	38.4 ± 0.05 ^h^
		SPK004/6/6	67.9 ± 0.12^ef^	51.6 ± 0.11^ef^
		Guntute	60.3 ± 0.12 ^g^	44.0 ± 0.11 ^g^
		Bucteca	53.4 ± 0.06^hi^	37.0 ± 0.05^hi^
		Kulto	54.3 ± 0.17^hi^	38.0 ± 0.10^hi^
CV (%)			2.91	3.39

Notes

CAC = CA concentration. Results are mean values of triplicate determination. Means with different letters across a column are significantly different.

#### 3.2.2 Total β‐carotene

The β‐carotene content in sweet potato can vary depending on the cultivars, harvesting conditions, maturity, and processing conditions (Van Hal, 2000). The β‐carotene content of flour samples in this study is within the range of 8.3–156.6 mg/100 g (Table 2) as reported for sweet potato flour (Dansby & Bovell‐Benjamin, 2003; Shih et al., 2009). The highest total β‐carotene contents (91.6 and 87.7 mg/100 g) were recorded from SPK004/6/6 and Kulto varieties, respectively, which were treated with 3% CA and dried at 55°C (Table 2). The lowest (33.5–38 mg/100 g) were from SPK00/06 and Guntute treated with 1% and dried at 65°C, and Kulto and Bucteca varieties treated with 3% CA but dried at 65°C. The recorded highest β‐carotene from SPK004/6/6 and Kulto varieties dried at 55°C and treated with 3% CA could be due to the fact that oxidation of β‐carotene at 55°C is minimal as compared to 65°C. The probable reasons for higher β‐carotene content from samples treated with 3% CA could be low activities of enzyme (peroxidases and lipoxygenases) at lower pH which have a capacity to degrade β‐carotene (Ahmed et al., 2011). Sharma, Kaur, Sarkar, Singh, and Singh (2009) also reported that β‐carotene loss was decreased at lower pH for carrot juice.

#### 3.2.3 Antioxidant activity potential and IC_50_ capacity

The ranges of values for percentage of antioxidant and IC_50_ of flour are shown in Table 3 as affected by variety, CA concentration, and drying temperature. The values (27.3–85.4%) are within the range of 9.7–99.6% as reported in Teow et al. (2007) for sweet potato flour. Varieties, CA concentration, and drying temperature showed significant differences in terms of antioxidant capacity. The results obtained revealed that varieties, CA concentration, and drying temperature influence the level of free radical inhibition of the flour. The highest percentages of inhibition (85.4 and 81.5%) were observed for SPK004/6/6 and Kulto varieties, respectively, which were treated with 3% CA and dried at 55°C. However, the lowest percentages of inhibition (27.3–31.9%) were observed for almost all varieties dried at 65°C (Table 3).

**Table 3:** Mean values of antioxidant capacity and IC_50_ values OFSP flour pretreated at different CA concentrations and dried at different temperatures for five different varieties

CAC	Drying temperature (°C)	Varieties	Inhibitions (%)	IC_50_ (mg/g)
CA (1%)	55°C	SPK00/06	39.0 ± 0.10 ^g^	11.3 ± 0.15^e^
		SPK004/6/6	57.5 ± 0.07^d^	1.9 ± 0.05 ^h^
		Guntute	48.5 ± 0.15^e^	3.3 ± 0.11 ^g^
		Bucteca	42.1 ± 0.16 ^fg^	9.2 ± 0.08 ^fg^
		Kulto	59.4 ± 0.08^d^	1.8 ± 0.11 ^h^
	65°C	SPK00/06	27.3 ± 0.15^i^	17.4 ± 0.15^a^
		SPK004/6/6	39.0 ± 0.15 ^g^	11.3 ± 0.10^e^
		Guntute	30.1 ± 0.14^hi^	15.1 ± 0.15^ab^
		Bucteca	32.4 ± 0.12 ^h^	14.2 ± 0.11 ^cd^
		Kulto	39.2 ± 0.16 ^g^	11.15 ± 0.08^ef^
CA (3%)	55°C	SPK00/06	76.4 ± 0.17^b^	1.08 ± 0.15^hi^
		SPK004/6/6	85.4 ± 0.15^a^	0.8 ± 0.10^i^
		Guntute	67.8 ± 0.14^c^	1.15 ± 0.12 ^h^
		Bucteca	75.2 ± 0.15^b^	1.1 ± 0.10^hi^
		Kulto	81.5 ± 0.16^a^	1.02 ± 0.10^hi^
	65°C	SPK00/06	32.3 ± 0.09 ^h^	14.3 ± 0.15^c^
		SPK004/6/6	45.4 ± 0.11^ef^	6.7 ± 0.15 ^g^
		Guntute	37.8 ± 0.14 ^g^	9.7 ± 0.09^f^
		Bucteca	30.8 ± 0.14^hi^	14.8 ± 0.11^b^
		Kulto	31.9 ± 0.13^hi^	14.4 ± 0.09^bc^
CV (%)			7.17	2.07

Notes

CAC = CA concentration. Results are mean values of triplicate determination, and means with different letters in a column are significantly different (*p* < 0.05).

The recorded highest values from SPK004/6/6 and Kulto varieties treated with 3% CA and dried at 55°C could be because of low oxidation of bioactive compounds at this temperature or because some of the antioxidants in flour were relatively not heat‐stable when the temperature increased to 65°C. This observation is in agreement with the work of Laine, Kylli, Heinonen, and Jouppila (2008), who reported that drying sweet tuber slices at a lower temperature results in greater antioxidant activity, but increasing drying temperature could degrade polyphenol compounds of the product and result in the loss of antioxidant activity. Chan, Lee, Yap, Aida, and Ho (2009) also stated that the loss in antioxidant capacities of plant product at high temperature is likely due to the degradation of polyphenols which were previously mobilized at low temperature. Similarly, Shahidi and Naczk (2003) reported that some polyphenols decomposed rapidly under high temperature and thus caused a reduction in the antioxidant capacity of plant sample. However, Lavelli et al. (1999) reported contradictory idea with the present study by stating that temperature releases more bound polyphenol compounds due to breakdown of cell wall phenolic compounds.

Data on the effects of drying temperature on polyphenol contents and antioxidant activity of vegetables are conflicting due to several factors, such as the drying method, type of extraction solvent, antioxidant assays used, and interactions of several antioxidant reactions. Another probable reason for the observed difference in terms of percentage of free radical inhibitions might be due to inactivation of polyphenol oxidase in acidic condition, which is the primary cause of the reduction in polyphenol contents. Retention of polyphenols results in an increase in the percentage of free radical scavenging ability. The treatment that has the highest antioxidant activity has also the highest concentration of bioactive compounds as indicated in Table 4 with a strong positive correlation. Higher positive correlation value with antioxidant capacity was observed for polyphenols as compared to β‐carotene and vitamin C contents. This might be based on the ability of the DPPH radical to react with hydrogen donor species, mainly polyphenols. Islam et al. (2002) and Kalaivani and Mathew (2010) also reported a strong positive correlation between antioxidant activity and polyphenol content. That is why polyphenols are very important plant constituents because of their scavenging ability on free radicals due to their hydroxyl groups (Tosun et al., 2009).

**Table 4:** Correlation values of total antioxidant capacity with total polyphenols, total β‐carotene, and vitamin C content

	Total polyphones	Total β‐carotene	Total antioxidant capacity
Total polyphenols	1	0.65 (<0.001)	0.88 (0.00)
Total β‐carotene		1	
Total antioxidant		0.76 (<0.001)	1

Note

The *p* values that show the significance of the correlations are shown in brackets.

The IC_50_ value, defined as the concentration of antioxidant required for 50% scavenging of DPPH radicals, is indicated in Table 3. The ranges of values for IC_50_ of the flour (0.8–17.4 mg/g) are comparable with the range of 1.7–17.4 mg/g reported for sweet potato flour (Teow et al., 2007). The highest values of IC_50_ (17.4 and 15.1 mg/g) were recorded from SPK00/06 and Guntute varieties, respectively, which were treated with 1% CA and dried at 65°C (Table 3). This could be due to loss of polyphenols during the drying process with increasing drying temperature and low concentration of CA applied. Islam et al. (2002) also stated that the temperature during drying affects the stability of polyphenols due to chemical and enzymatic degradation which cause a reduction in polyphenol contents. Many studies reported that high polyphenol content contributes to high radical scavenging activity (Islam et al., 2002; Teow et al., 2007).

## 4 CONCLUSION

Production of sweet potato is seasonal, whereas consumption is all year round. As a perishable product, the tuber cannot be stored for long period of time and experience high after‐harvest loss. In line with good postharvest handling, value addition through change of form of the product is a strategy to reduce losses, diversify the product, and to store it for reasonable time under proper storage conditions. OFSP evaluated in this study showed different responses in terms of nutrient contents, bioactive components, and total antioxidant capacity. Treatment of OFSP slices in 3% CA solution after drying at 55°C significantly preserved better nutrients and bioactive components with better antioxidant capacities as compared with 1% CA solution and drying temperature of 65°C. The lower the temperature with 3% CA concentration contributes for better preservation effect. Among the evaluated varieties, at selected drying temperatures and CA solution concentrations, Kulto and SPK006/6/6 performed better for most of the parameters studied, followed by SPK00/06. This implies that these three varieties are more tolerant of drying medium heat and low pH effect. Therefore, under these selected conditions, value‐added dried OFSP can be produced for use as an ingredient to make diverse foods for different social groups besides its contribution to the avoidance of after‐harvest losses.

## ETHICAL STATEMENT

This study did not involve any human or animal testing.

## CONFLICT OF INTEREST

All authors declare no conflict of interest.

## AUTHORS' CONTRIBUTIONS

The first author was responsible for the accomplishment of most of the laboratory experiments with the help of the second author. All the authors listed in this manuscript contributed equally to the preparation of the manuscript and approved it for publication.
